# Annotations

**Published:** 1906-07-28

**Authors:** 


### ANNOTATIONS.

### Boron Preservatives in Tinned Ham.

The question of boron preservatives in food was carefully inquired into by a Departmental Committee, whose report was published some five years ago. The report is to-day the most reliable source of information upon this subject. In view of this fact we must express surprise at the successful result of the recent prosecution of Messrs. Richard Dickeson and Company, Limited, the well-known Army contractors. The Brentford Bench of Magistrates recently held that the tinned ham of this firm was injurious to health because it contained fifty-two grains of boracic acid to the pound. It transpired in evidence that some 700,000 of these tins of potted ham had been sold by this firm in military canteens, and that no complaint against the article had ever been made. It also transpired in evidence that the Preservatives Committee, from an exhaustive examination of hams, discovered that some 90 per cent. of all hams on the market contained boracic acid, some in as large a quantity as forty-six grains to the pound. Further, from an examination of the amount of boracic acid in this class of foods—namely, potted meats—the Committee became aware that some samples contained as much as sixty-six grains of boracic acid to the pound. With these facts before them they state distinctly in their report, although in certain classes of food they entirely prohibit the use of all chemical preservatives, that they see no reason for interfering with the use of boron preservatives in cured and potted meats. They give as the chief reasons for this that the public will no longer buy highly salted foods, and that if salt is not used some other chemical preservative must take its place, and that in the consumption of foods of this class, which are only taken in relatively small quantities, a very small amount of the antiseptic is introduced into the system. The potted ham in question was sold in tins weighing approximately 2 oz. and costing 1d. It was really used more as a condiment than as a food, and probably the maximum amount consumed by any one individual in one day would be half a tin, or, in other words, under two grains of boracic acid. It was seriously held by the magistrates that this quantity of boracic acid would either injure the health of the British soldier or his children. There is a large demand for potted meats, either tinned or preserved in glass, and about 75 per cent. of these contain boracic acid; their popularity depends, especially amongst the poorer classes, upon the fact that they will keep not only before they are opened, but afterwards, and it is to ensure the keeping after opening that this chemical preservative is used. The poorer classes cannot afford to throw away food. The choice often is, then, between bad food and preserved food, and all are agreed that boracic acid is the least injurious of all chemical preservatives. In connection with food legislation Timon's words may usefully be recalled. When Timon had made his laws the Athenians, being disappointed, asked him if those were the best laws he could make. "No," said Timon, "they are not; but they are the best laws you can keep." While this article was going through the press a question was asked in the House of Commons about the Dickeson case. It teaches a useful lesson with regard to the attempt which appears to be perseveringly made to prejudice the sale of any kind of tinned food. The meat was described by the questioner as putrid; if the report of the case had been read, this gentleman would have known that it was never alleged by the prosecution that there was anything whatever wrong with the meat, the only objection to the product being the presence of the preservative in the above quantity.

### The Pharmacopœial Standard.

A case has recently been decided in one of the Scottish courts which raises a delicate point in reference to the claim of the Pharmacopœia to be the legal standard for medicines described in its pages. An inspector under the Sale of Food and Drugs Act purchased from a chemist, for purposes of analysis, a sample of liquid extract of cascara sagrada. At the time of the sale the seller explained that the article supplied, though of the official strength, was made by a special process and did not in every respect conform to the pharmacopœial standard. On analysis it was found that the preparation did not contain alcohol, but some other preservative agent, and consequently the chemist was summoned to appear before the Sheriff and charged with the sale of an article not of the nature and quality demanded, and to the prejudice of the purchaser. The court found that as a matter of fact the inspector knowingly accepted the article supplied to him for what it actually was and pretended to be, and therefore held that the purchaser had not been "prejudiced." Such a conclusion in this particular case was no doubt just, as well as inevitable, but it is advisable to emphasise the contention that when a medicine for which a formula is supplied in the Pharmacopœia is ordered, that formula must be followed in every particular. It is not sufficient to say that the essential ingredient, both in quantity and quality, satisfies the official claims. All other ingredients must also satisfy these demands, and no substitution or alteration can for a moment be defended. The provision of "one uniform standard and guide" for the preparation and dispensing of medicines is the main object of a national Pharmacopœia; and individual alterations, however slight, are contrary to the essential principle of the volume. This position is, we believe, clearly recognised by pharmacists as vital to the performance of their dispensing responsibilities.

### West London Hospital Vacation Course.

A short vacation course of 24 lectures has been arranged at the Post-Graduate College, to begin on Monday, August 13, 1906. The college has excellent facilities for practical instruction. The lecturers and demonstrators include Dr. Low, Dr. Davis, Dr. Russel, Dr. Abraham, Dr. Seymour Taylor, Mr. Paget, Mr. Dunn, Mr. Armour, Mr. Pardoe, Mr. Bidwell, and Mr. Chisholm Williams.

